# Antimicrobial Resistance in Maternal Infections During Pregnancy

**DOI:** 10.3390/biomedicines13040777

**Published:** 2025-03-22

**Authors:** Tania Vlad, Anca-Elena Eftenoiu, Adela Voinescu, Silvia Ioana Musuroi, Corina Musuroi, Aurica Elisabeta Moatar, Daliborca Cristina Vlad, Adriana Trandafir, Cristian Sebastian Vlad, Ionut Marcel Cobec

**Affiliations:** 1 Doctoral School, Faculty of Medicine, “Victor Babes” University of Medicine and Pharmacy, 300041 Timisoara, Romania; 2 Department of Medical Genetics, “Carol Davila” University of Medicine and Pharmacy, 050474 Bucharest, Romania; 3 Microbiology Department, Multidisciplinary Research Center of Antimicrobial Resistance, “Victor Babes” University of Medicine and Pharmacy, 300041 Timisoara, Romania; 4 Emergency County Clinical Hospital Pius Brinzeu Timisoara, 300723 Timisoara, Romania; 5 ANAPATMOL Research Center, Faculty of Medicine, “Victor Babes” University of Medicine and Pharmacy, 300041 Timisoara, Romania; 6 Clinic of Internal Medicine-Cardiology, Klinikum Freudenstadt, 72250 Freudenstadt, Germany; 7 Department of Pharmacology, Faculty of Medicine, “Victor Babes” University of Medicine and Pharmacy, 300041 Timisoara, Romania; 8 “Bagdasar-Arseni” Emergency Clinical Hospital, 050474 Bucharest, Romania; 9 Clinic of Obstetrics and Gynecology, Klinikum Freudenstadt, 72250 Freudenstadt, Germany

**Keywords:** infections, pregnancy, newborns

## Abstract

**Background**: An imbalance in the vaginal microbiota, often characterized by reduced lactobacilli, paves the way forth for opportunistic bacteria from the gastrointestinal tract. The presence of aerobic bacteria in the genital tract during pregnancy can have negative outcomes on the pregnancy. Peripartum infections, when not adequately managed, can significantly impact maternal and neonatal health. Antimicrobial resistance poses an escalating global health threat, with newborns particularly vulnerable. **Methods**: This study constitutes a retrospective observational analysis, encompassing all microbial strains isolated from pregnant women admitted to the “Pius Brînzeu” Clinical County Emergency Hospital in Timișoara, Romania for various infectious diseases over one year. We analyzed 274 samples from 246 pregnant women, of which 242 were cervical samples, 23 urine cultures, 3 wound secretions, 3 amniotic fluids, 1 peritoneal cavity fluid, 1 sputum, and 1 hemoculture. **Results**: In cervical samples, Group B Streptococcus (GBS) was the most prevalent, representing 42.46% of the isolates. *E. coli* was the second most frequent at 30.16%, followed by *K. pneumoniae* at 11.9%, *S. aureus* at 8.73%, *C. albicans* at 2.78%, and other species at 3.97%. A total of 9.63% of cervical GBS isolates exhibited resistance to penicillin, while 23.36% were identified as multi-drug resistant (MDR). Methicillin-resistant *S. aureus* (MRSA) and MDR *S. aureus* strains were identified in 50% and 54.54% of the *S. aureus*-positive cervical samples, respectively. **Conclusions**: Recognizing the implications of maternal infection or colonization, especially with antimicrobial resistance bacteria, aids in assessing risks during pregnancy.

## 1. Introduction

In recent years, more attention has been directed toward the vaginal microbiome and the implications of its imbalance on women’s health and especially on maternal-fetal health. Research has shown that, in areas of the body with high bacterial colonization, the occurrence of clinical infection is not due to an increase in the concentration of pathogenic microorganisms, but rather to a decrease in the concentration of beneficial commensals—a state known as dysbiosis [1]. Sometimes, fluctuations in the vaginal microbiome are caused by age, the body’s hormonal status, sexual activity, behavioral and hygiene habits, immune status, and physiological changes such as pregnancy [2]. The vaginal microbiota plays an important role during the implantation of the embryo, and dysbiosis is often associated with infertility [3]. Dysbiosis, if left untreated, can lead to gynecological pathology (such as infections of the genital tract), pregnancy loss, preterm labor, and reduced conception rates [4,5,6]. The hormonal and mechanical changes in the female genital tract in pregnancy increase the risk and severity of vaginal infections. These infections can lead to miscarriage and even recurrent spontaneous miscarriage, stillbirth, preterm birth, and the transmission of infections to the fetus [7]. The vaginal microbiota is dominated by *Lactobacillus* species, with a key role in the control of the vaginal pH. During pregnancy, a notable decrease in the diversity and stability of the vaginal microbiome has been observed, with a predominance of *Lactobacillus* spp., *Actinomycetales*, *Clostridiales*, and *Bacteroidales* [2,8]. Pathogenic agents have the potential to alter the normal physiological vaginal discharge, leading to conditions such as vaginitis, cervicitis, or pelvic inflammatory disease. Common causes include bacterial vaginosis, vulvovaginal candidiasis, trichomoniasis, *Chlamydia trachomatis*, and *Neisseria gonorrhoeae* infections. Those infections can lead to long-term consequences or long-term sequelae, such as an increased risk of ectopic pregnancy, infertility, or chronic pelvic pain [9].

Aerobic vaginitis (AV), one of the most frequent female reproductive tract infections, differs from the well-known bacterial vaginosis (BV) in two main aspects. One of them is the origin of the causative microorganisms—while bacterial vaginosis is an endogenous infection, aerobic vaginitis is caused by bacteria of intestinal origin, superimposed on a dysbiosis of the vaginal microenvironment that normally consists of commensal aerobic microorganisms of intestinal origin: *S. agalactiae* (group B Streptococcus, GBS), *E. coli*, *S. aureus*, *K. pneumoniae,* and *E. faecalis*. A noteworthy aspect is that a positive vaginal culture for the aforementioned microorganisms is not sufficient for the diagnosis of AV. The second main difference between the two types of infections is that aerobic vaginitis is associated with more inflammation [10].

The main microorganisms involved in aerobic vaginitis are consistent worldwide, though the prevalence of individual bacteria varies by region. An Indian study from 2021 found that 15% of patients presenting with abnormal discharge had AV, the causative agents being *E. coli* (7.5%), *S. aureus* (4.5%), *Klebsiella* spp. (2%), and *Enterococcus* spp. (1%). Another Indian study conducted on low-risk asymptomatic pregnant women found that 52.6% had vaginal colonization with pathogenic aerobic bacteria, among which 13.2% were colonized with coagulase-negative *S. aureus* and 8.9% with *E. coli*. A study conducted in Poland on patients with clinical signs of vaginal infection revealed that the most frequently isolated species in vaginal swabs was *E. faecalis* (29.2%), followed by *E. coli* (26.3%), and *S. agalactiae* (13.1%) [10,11,12].

Studies have reported that up to 49% of AV cases are asymptomatic, but regardless of clinical manifestation, these infections were associated with recurrent pregnancy loss. One study showed that the prevalence of AV in patients with previous recurrent pregnancy loss (RPL) was almost six-fold higher than its prevalence in pregnant women without RPL (70.7% as opposed to 12%) [7].

AV is treated with antibiotics with intrinsic activity against aerobic bacteria. The United States Food and Drug Administration (FDA) developed a labeling system (ABCDX) that designates the safety of a drug for use during pregnancy. Clindamycin is a broad-spectrum antibiotic active against aerobic Gram-positive and anaerobic bacteria. As a category B drug, it is commonly used in the treatment of aerobic vaginitis during pregnancy, reducing the incidence of infection-related premature delivery and other adverse pregnancy outcomes. An in vitro study on 1923 vaginal swabs from aerobic vaginitis patients determined that carbapenem and β-lactam–β-lactamase inhibitor combinations were the most effective antibiotics; these combinations are also labeled as category B, so they can be considered in pregnancy [10].

Although AV is an important cause of pregnancy complications, antibiotic therapy may not be the optimal approach to positive vaginal culture results, since this leads to the selection of antibiotic-resistant bacteria (ARB). The recent literature highlights the importance of an alternative approach that shifts the focus from decreasing pathological microorganisms toward increasing beneficial ones. This approach is based on probiotics and immune modulators (for example, local estrogen or progesterone), which could be associated with targeted antibiotic treatment or employed as an alternative therapy. Studies have demonstrated the effectiveness of different non-antibiotic approaches for prophylaxis of AV recurrence—combined intravaginal probiotic and low-dose estriol or silver nanoparticles active against *E. coli* and *S. aureus* biofilms [7,10].

The World Health Organization (WHO) defines maternal peripartum infections as symptomatic bacterial infections of the genital tract or surrounding tissues that occur at any time between the onset of rupture of membranes or labor and the 42nd day following childbirth. While antibiotics are the predominant strategy for prevention and treatment, their misuse during pregnancy is widespread, contributing to antimicrobial resistance. This facilitates the emergence of multi-drug resistant (MDR) strains, defined by resistance to at least one antibiotic in three or more classes. The WHO recommends antibiotics for women with the preterm labor rupture of membranes, intrapartum antibiotics for women with GBS colonization to prevent neonatal infection, and routine antibiotic prophylaxis for women undergoing cesarean section or manual placenta removal, or with perineal tears that extend to the anal sphincter or the anal canal [13].

Current strategies for antibiotic use during pregnancy include antibiotic treatment for confirmed infections, symptomatic bacterial vaginosis, asymptomatic bacteriuria to prevent pyelonephritis, and antepartum GBS colonization [14,15,16].

This study aims to analyze the microorganisms responsible for maternal infections and their associated antimicrobial resistance patterns, with a focus on infections or colonization of the genito-urinary tract.

## 2. Materials and Methods

This study constitutes a retrospective observational analysis, encompassing all microbial strains isolated from pregnant women admitted to the “Pius Brînzeu” Clinical County Emergency Hospital in Timișoara, Romania, for various infectious diseases. The time frame for this analysis extends from 1 January 2023 to 31 December 2023, covering a period of one year.

All the bacterial strains included in this study were isolated in pure culture or in significant quantities. The microscopic examination, of Gram-stained smears, revealed the presence of the inflammatory reaction and the frequency of bacteria.

Microbial identification was performed with the use of Matrix-Assisted Laser Desorption/Ionization–Time-of-Flight (MALDI-TOF) mass spectrometry for identifying microorganisms. Antimicrobial sensitivity tests used the VITEK automated microbiology system with Minimum Inhibitory Concentration (MIC) determination, in accordance with the 2021 Clinical and Laboratory Standards Institute (CLSI) guidelines. The Unyvero molecular diagnostics system was used for gene identification in cases of newborn bloodstream infections.

Over the above-mentioned period, we received 274 samples from 246 pregnant women who were either being monitored for maternal diseases (hypertension, kidney diseases, thyroiditis, and anemia) or had pregnancy-related disorders (for example, preeclampsia or placenta praevia).

Data collection, analysis, and visualization were performed using Microsoft Excel 2019.

## 3. Results

The study group consisted of 246 pregnant women from whom a total of 274 samples were analyzed. Of the 274 samples, 242 were cervical samples and the remaining 32 were non-cervical, as follows: 23 urine cultures, 3 wound secretions, 3 amniotic fluids, 1 peritoneal cavity fluid, 1 sputum, and 1 hemoculture (Table 1).

### 3.1. Non-Cervical Samples

A total of 32 non-cervical samples were studied and all were monobacterial. Table 1 describes the microorganisms identified in these samples, along with their antimicrobial resistance (AMR) patterns. *E. coli* was identified in 15 of the 23 urine samples, with one case exhibiting a cephalosporinase-producing phenotype, one MDR strain exhibiting resistance to fluoroquinolones (R-FQ) and trimethoprim-sulfamethoxazole (R-SXT), and one R-SXT strain. Two urine samples were positive for *K. pneumoniae*, one pan-drug-resistant, i.e., resistant to all tested antibiotics, and the other one an MDR strain, extended spectrum beta-lactamase (ESBL)-producing and R-FQ. The other samples (five positive for *Enterococcus* spp. and one for *S. saprophyticus*), exhibited isolated antimicrobial resistances, without therapeutic implications.

Analysis of the amniotic fluid samples demonstrated the presence of a single isolate of each of the following microorganisms: *S. epidermidis*, *K. pneumoniae*, and *Enterococcus faecalis*. These isolates were characterized by an absence of acquired antimicrobial resistance phenotypes.

Wound secretions displayed an MDR *S. aureus* strain resistant to methicillin, fluoroquinolones, and trimethoprim-sulfamethoxazole; an R-FQ, R-SXT *E. coli* strain; and a strain pertaining to *Klebsiella pneumoniae* without relevant antimicrobial resistance.

The single sample of peritoneal cavity fluid presented *S. anginosus* sensitive to all tested antibiotics.

The hemoculture was positive for *E. coli*, without any relevant antimicrobial resistance.

A strain of *K. pneumoniae* presenting extended drug resistance (XDR) was isolated from the sputum sample.

### 3.2. Cervical Samples

**Figure 1:**
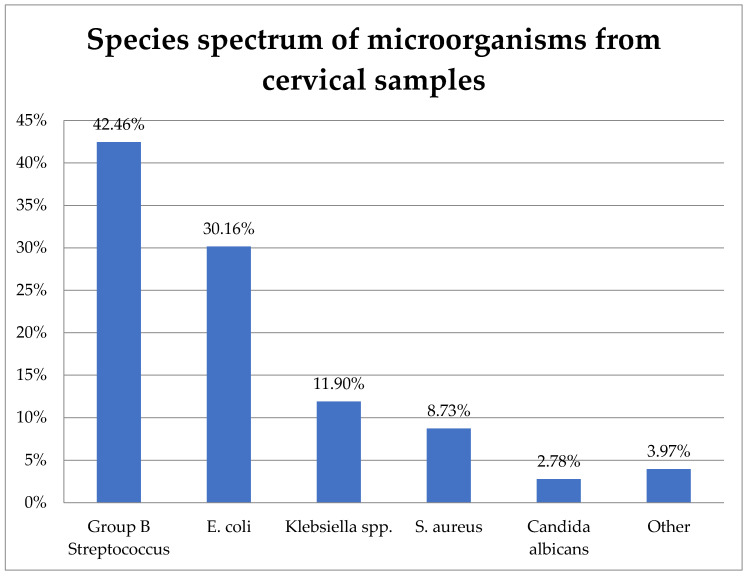
Species spectrum of microorganisms from cervical samples.

The study included 242 cervical samples from which 252 bacterial strains were isolated. Their distribution is shown in Figure 1.

GBS (*S. agalactiae*) was the most prevalent, representing 42.46% (107) of the isolates. *E. coli* was the second most frequent at 30.16% (76), followed by *K. pneumoniae* at 11.9% (30), *S. aureus* at 8.73% (22), *C. albicans* at 2.78% (7), and other species at 3.97% (10).

### 3.3. Group B Streptococcus

Out of the 107 cervical samples with GBS, 62.62% (67) were from monitored pregnancies or pregnancies that had reached full-term, 23.36% (25) from abortion, 8.41% (9) from premature birth, 4.67% (5) with pregnancy-related disorders, and 0.94% (1) from ectopic pregnancy. The five cases with pregnancy-related disorders included three instances of placenta praevia, one of cervico-isthmic insufficiency, and one of pregnancy cholestasis (Table 2).

**Figure 2:**
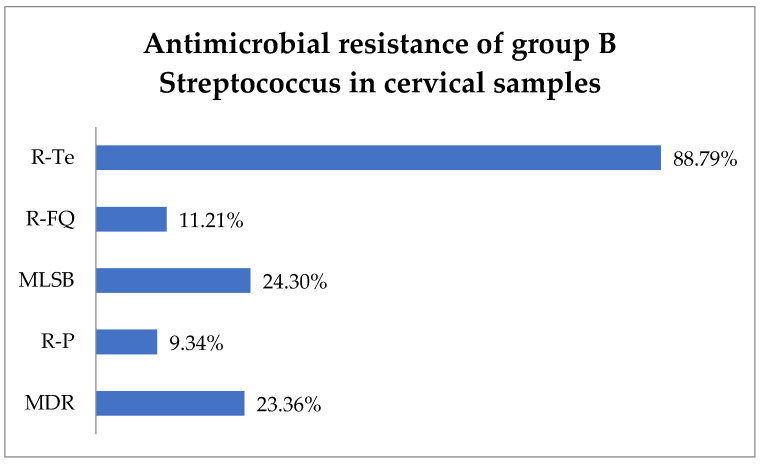
Antimicrobial resistance of group B Streptococcus in cervical samples. Abbreviations: R-Te = resistance to tetracycline, R-FQ = resistance to fluoroquinolones, MLSB = macrolide–lincosamide–streptogramin B, R-P = resistance to penicillin, MDR = multi-drug resistant.

Antimicrobial susceptibility testing revealed that 88.79% (95) of GBS isolates exhibited resistance to tetracycline (R-Te), 11.21% (12) to R-FQ, and 9.34% (10) to penicillin (R-P). Macrolide–lincosamide–streptogramin B (MLSB) resistance was observed in 24.3% (26) of isolates, while MDR was observed in 23.36% (25) (Figure 2).

### 3.4. E. coli

Regarding the 76 samples positive for *E. coli*, 15.79% (12) pertained to cases of abortion and 14.47% (11) to cases of premature birth. A total of 6.58% (5) of *E. coli*-positive cervical samples were from ectopic pregnancies. Maternal diseases accounted for 6.58% (5) of *E. coli*-positive cervical samples, including three cases of urinary tract infection (UTI), one case of hypothyroidism, and one case of heart failure, while 3.95% (3) accounted for pregnancy-related disorders, including two cases of placenta praevia and one case of dysgravidia. In total, 52.63% (40) of cases with *E. coli*-positive cervical samples were from monitored or full-term pregnancies (Table 2)

**Figure 3:**
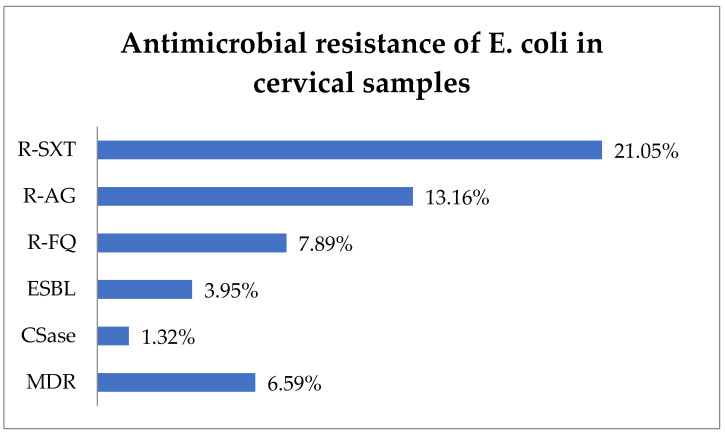
Antimicrobial resistance of *E. coli* in cervical samples. Abbreviations: R-SXT = resistance to trimethoprim–sulfamethoxazole, R-AG = resistance to aminoglycosides, R-FQ = resistance to fluoroquinolones, ESBL = extended spectrum beta-lactamase, CSase = cephalosporinase, MDR = multi-drug resistant.

R-SXT was identified in 21.05% (16) of *E. coli* isolates, R-AG in 13.16% (10), and R-FQ in 7.89% (6) (Figure 3). The ESBL phenotype represented 3.95% (3) of cases, while the CSase phenotype represented 1.32% (1). In total, 6.59% (5) of *E. coli* strains exhibited an MDR phenotype.

### 3.5. K. pneumoniae

Out of 30 cases where *K. pneumoniae* was isolated in cervical samples, 16.67% (5) were associated with abortion, 16.67% (5) with premature birth, and 3.4% 3.33% (1) with ectopic pregnancies. In total, 63.33% (19) of the *K. pneumoniae*-positive samples were from ongoing or full-term pregnancies (Table 2).

**Figure 4:**
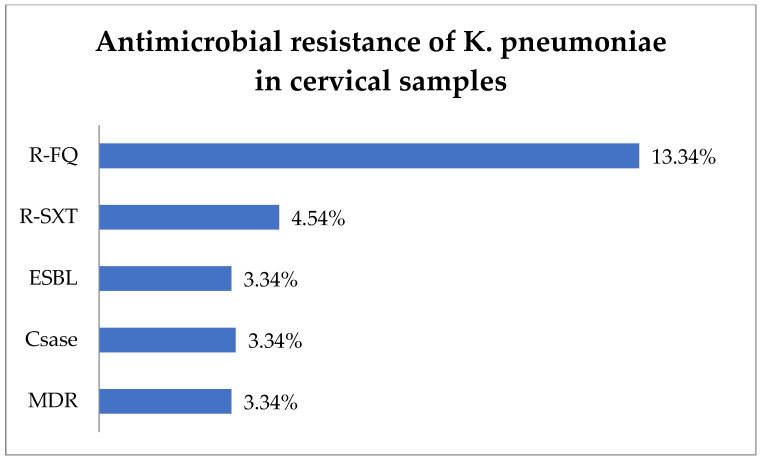
Antimicrobial resistance of *K. pneumoniae* in cervical samples. Abbreviations: R-FQ = resistance to fluoroquinolones, R-SXT = resistance to trimethoprim–sulfamethoxazole, ESBL = extended spectrum beta-lactamase, CSase = cephalosporinase, MDR = multi-drug resistant.

The antibiotic resistance testing of the *K. pneumoniae* isolates (30) from cervical samples showed that R-FQ was present in 13.34% (4) of the isolates, while R-SXT was in 4.54% (1 of 22 tested) (Figure 4). Equal rates of 3.34% (1) each were noted for ESBL and Csase phenotypes. MDR was found in 3.34% (1) of the *K. pneumoniae* isolates.

### 3.6. S. aureus

Analysis of 22 cervical samples positive for *S. aureus* revealed that 40.9% (9) were in the context of uncomplicated monitored or full-term pregnancy, while 31.82% (7) were associated with abortion, 18.18% (4) with premature birth, 4.55% (1) with pregnancy-related disorders represented by dysgravidia, and 4.55% (1) with maternal disease represented by heart failure (Table 2).

**Figure 5:**
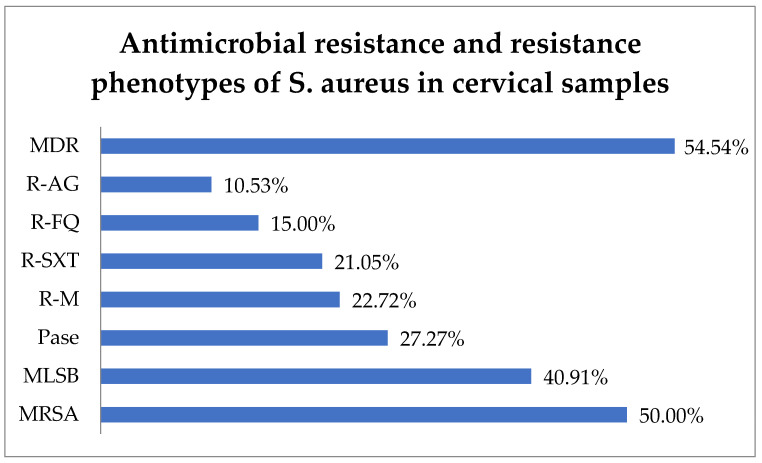
Antimicrobial resistance of *S. aureus* in cervical samples. Abbreviations: MDR = multi-drug resistant, R-AG = resistance to aminoglycosides, R-FQ = resistance to fluoroquinolones, R-SXT = resistance to trimethoprim–sulfamethoxazole, R-M = resistance to macrolides, Pase = penicillinase, MLSB = resistance to macrolide–lincosamide–streptogramin B, MRSA = methicillin-resistant Staphylococcus aureus.

Antibiotic resistance testing indicated that 50.0% (11) of *S. aureus* isolates were identified as methicillin-resistant *S. aureus* (MRSA) and 40.91% (9) exhibited resistance to macrolide–lincosamide–streptogramin B (MLSB). In total, 27.27% (6) of *S. aureus* strains produced penicillinase (Pase). Additionally, resistance to macrolides (R-M) was observed in 22.72% (5) of isolates, R-SXT in 21.05% (4), R-FQ in 15.0% (3), and R-AG in 10.53% (2). In this context, 54.54% (12) of the strains were found to have MDR and 13.63% (3) difficult-to-treat resistance (DTR), i.e., they were resistant to all first-line agents (Figure 5).

**Figure 6:**
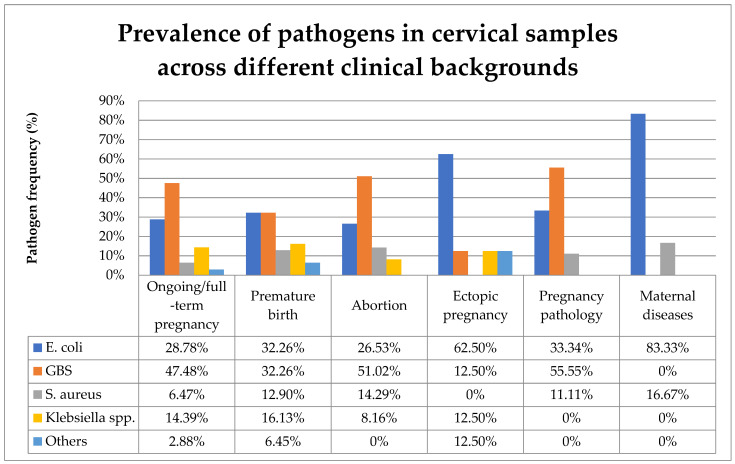
Prevalence of pathogens in cervical samples across different clinical backgrounds. Abbreviations: GBS = group B Steptococcus.

Figure 6 compares the prevalence of the various pathogens identified in cervical samples across different clinical backgrounds.

## 4. Discussions

Maternal infections are a major concern since they can lead to severe complications for both the mother and the newborn. Approximately half of early-preterm births (<28 weeks of gestation) and the majority of early-onset cases of neonatal sepsis are attributed to maternal infection during pregnancy [17]. A data analysis of nearly 3 million live births across 52 countries reveals that severe maternal infection during pregnancy is strongly associated with stillbirth and early neonatal death [17]. Infectious diseases contribute to approximately 23% of neonatal deaths, including 15% from neonatal sepsis [18]. Recent years have witnessed an increase in the prevalence of antibiotic-resistant bacteria as causative agents of early- and late-onset sepsis in neonates [19]. Early-onset neonatal infections can be vertically acquired from bacteria colonizing the mother’s reproductive tract and, despite being asymptomatic for the mother, these bacteria could potentially cause illness in the newborn [13].

Studies differing in protocol type, sample size, region, and time period have indicated that between 25% and 40% of premature births are caused by intrauterine infection. Multiple studies indicate that preterm labor is due to a decrease in lactobacilli, rather than an increase in pathological microorganisms. In light of these findings, it has been hypothesized that the concentration of vaginal Lactobacillus spp. can be a clinical tool to forecast the risk of preterm labor [20].

Regarding maternal outcomes, in addition to increased morbidity and mortality, persistent pelvic discomfort, fallopian tube obstruction, and secondary infertility are among the long-term impairments that women who suffer from peripartum infections are susceptible to [13].

In the present study, the majority of cervical samples positive for bacteria were observed during uncomplicated ongoing or full-term pregnancies. GBS was the most prevalent bacteria found in the cervical samples, followed by *E. coli*, *Klebsiella pneumoniae*, and *S. aureus*. The presence of diverse aerobic bacteria in the genital tract varies across different obstetrical backgrounds. In ongoing or full-term pregnancies, the most frequently identified pathogen in cervical samples was GBS. Among premature birth cases, *E. coli* and GBS were equally prevalent. GBS was present in the cervical samples of more than half of both abortions and pathological pregnancies. In cases of ectopic pregnancy and maternal diseases, *E. coli* was the most prevalent cervical pathogen (Figure 6).

*S. agalactiae* is a normal microbiota constituent present in 11.1 to 22.4% of healthy adults (with regional differences) and 7 to 25% of women between 35 and 37 weeks of gestation with aerobic vaginitis. However, GBS colonization in pregnancy is associated with premature birth, chorioamnionitis, and stillbirth, and GBS infection is a worldwide leading cause of neonatal sepsis and meningitis. The highest incidence of GBS infection has been found in neonates and infants younger than 3 months. It has been estimated that in 2015 GBS infection caused 319,000 cases of neonatal disease, resulting in 90,000 deaths and more than 57,000 stillbirths [21].

According to the Center for Disease Control and Prevention (CDC), GBS represents the primary infectious cause of infant death in the United States of America [22]. A systematic review in 2017 estimated that 20.8% (95%CI, 17.3–24.4) of pregnant women in Eastern Europe present lower gastrointestinal and genital tract colonization with GBS [23]. In order to decrease the incidence of newborn GBS infection, the screening of pregnant women for this organism in the third trimester has been implemented, followed by antibiotic prophylaxis in case of maternal colonization. Neonatal infection is associated with maternal colonization at the time of delivery, predominantly through vertical transmission [24].

The American College of Obstetricians and Gynecologists (ACOG) recommends antepartum screening for GBS between 36 + 0 and 37 + 6 weeks of gestation [25]. Intrapartum antibiotic prophylaxis with a beta-lactam antibiotic administered at least four hours before delivery is recommended for women with positive GBS screening results [25]. In our study, 24.3% presented MLSB resistance phenotype, while 23.4% were identified as MDR. More than 80% of GBS strains were resistant to tetracycline, which is consistent with the rates recently reported in the literature [26]. These antimicrobial resistances pose challenges for both intrapartum prophylaxis and neonatal infection treatment.

We observed a high prevalence of β-hemolytic *Streptococcus* in ongoing or full-term pregnancies (47.5%) and in women admitted for premature birth (32.3%). The presence of GBS in these cases necessitates adequate monitoring and treatment due to its potential to cause disease in newborns. Moreover, more than 50% of abortion cases presented β-hemolytic *Streptococcus* in cervical samples.

In our study, *E. coli* was the second most prevalent microorganism in cervical secretions, but given the likelihood of cervical contamination and the antibiotic susceptibility indicative of community-acquired strains, treatment should only be administered if clinically indicated. Treatment for *E. coli*-positive cervical secretions is necessary only if accompanying symptoms are present. Even in asymptomatic vaginosis in the mother, the antimicrobial resistance patterns of the colonizing bacteria are important in case early infections occur in newborns.

Notably, a high percentage of *E. coli* strains, 21.1%, exhibited resistance to sulfonamides.

*E. coli* vaginal colonization affects 32% (95%CI, 17–48%) of pregnant women, according to a systematic review of 2021 [27]. Vaginal infection with *E. coli* is associated with the premature rupture of membranes, premature delivery, abortion, stillbirth, and other negative pregnancy outcomes [10]. Most extraintestinal *E. coli* infections are susceptible to a range of antibiotics, such as beta-lactam antibiotics, fluoroquinolones, trimethoprim–sulfamethoxazole, nitrofurantoin, and fosfomycin [28].

*Klebsiella* spp. was identified in 11.9% of the cervical samples acquired in the present study. Notably, certain strains of *Klebsiella* exhibited MDR and ESBL antimicrobial resistance patterns. However, these resistance patterns were observed in isolated cases and did not pose significant therapeutic challenges.

A study of the vaginal microbiota of pregnant women showed that an increase in *K. pneumoniae* levels in the vagina was an independent risk factor for preterm delivery. Another study showed that *K. pneumoniae* increases the risk of chorioamnionitis, the risk of respiratory distress syndrome, and the risk of patent ductus arteriosus [10,29,30].

Among the four primary bacteria identified in cervical samples in our study, *S. aureus* was found to be the least prevalent.

Infants born to mothers who have nasal or vaginal *S. aureus* colonization are susceptible to acquiring *S. aureus*, with horizontal transmission from the mother likely being the primary source of *S. aureus* in newborns [31]. *S. aureus* infection is identified in over 90% of cases of late-onset sepsis in neonates and late-onset sepsis caused by *S. aureus* occurs four times more often in very-low-birth-weight infants than in normal-weight infants [10].

Maternal vaginal colonization with MRSA may lead to neonatal infection through vertical transmission [32]. The present study identified MRSA and MLSB-R *S. aureus* in 50% and 40.9% of *S. aureus*-positive cervical samples, respectively. Moreover, more than half of *S. aureus* strains were MDR, and three strains were resistant to all first-line agents. According to the European Centre for Disease Prevention and Control, MRSA rates in Romania are among the highest in Europe, with 25 to < 50% of *S. aureus* invasive isolates being resistant to methicillin [33].

From the studied non-cervical samples, a quarter of the identified strains presented relevant resistance to antibiotics. Three of these strains were MDR, one was PDR, and one was XDR.

The predominant strain found in the studied urine samples was *E. coli*, aligning with data reported previously [34]. The presence of bacteria in urine samples causes either asymptomatic bacteriuria or urinary tract infection (UTI). Pregnancy promotes the progression of UTI, potentially resulting in negative outcomes for both the mother and the fetus, therefore pregnant women should be routinely screened for asymptomatic bacteriuria [35].

Febrile UTIs are the most common bacterial infections in children, with their severity inversely correlated with the age of the child. *E. coli* and *Klebsiella* spp. are the most common causes of UTIs in children, so the first line of antibiotic treatment is beta-lactams. Recent years have seen an increase in the prevalence of UTIs caused by ESBL-producing bacteria; of note is the fact that this finding occurred even in infants rarely exposed to antibiotics. This prompts the hypothesis that the maternal use of antibiotics may increase the risk of the infants developing an ESBL-positive UTI—possibly through intestinal colonization with resistant bacterial strains from the mother [35].

However, this transmission need not always be hospital-associated—an epidemiological study that analyzed ESBL transmission rates in hospital vs. household settings discovered a predominance of household transmission over nosocomial transmission [36].

A systematic review published in 2016 showed that the global rate of UTIs with ESBL-producing microorganisms was 14% [37].

In a recent retrospective study, after controlling for confounding factors such as delivery mode, prematurity, or feeding type, no significant differences were revealed between ESBL-positive and ESBL-negative groups. This could mean that the protective factors against developing a UTI are not protective factors against ESBL positivity in UTIs. The same study revealed that antibiotic treatment of the mother during pregnancy was the only modifiable risk factor for community-acquired UTI caused by ESBL-producing bacteria, with *Klebsiella* spp. being two-fold more highly transmitted than *E. coli*. Therefore, the antibiotic exposure of the mother during pregnancy should be taken into account in the management of UTIs in the infant [35].

Colonization with ESBL-producing bacteria has been hypothesized as a cause of severe pregnancy and neonatal problems, among which preterm delivery and neonatal infections—the transmission of ESBL from the mother—increases the risk of the neonate developing early- or late-onset neonatal sepsis. Newborns from colonized mothers are six times more likely to be colonized as well, maternal ESBL colonization being considered the most important risk factor for the colonization of very-low-birth-weight infants, with reported mother-to-child transmission rates of 27.8%, 36%, and even as high as 43% [38]. Although colonization does not directly reflect infection rates, maternal colonization with ARB is a risk factor for colonization in newborns [19]. It has been shown that neonatal colonization patterns can persist for up to five years, with longer persistence of the more resistant strains [39].

Although it is possible that other factors contributed to the high rate of preterm births in ESBL-positive groups of pregnant subjects, the association between the vaginal presence of these bacteria and preterm birth is clear, suggesting the need for routine screening in at-risk individuals [38].

Antibiotic exposure in pregnancy is associated with a higher risk of pediatric infection-related hospitalizations, very-early-onset inflammatory bowel disease, and asthma (especially in the case of cephalosporins and macrolides), with a dose-dependent increase in risk for asthma. The risk of childhood asthma has been proven to be higher in children exposed prenatally to broad-spectrum antibiotics than in those exposed to narrow-spectrum antibiotics. A prospective birth cohort study on 1080 European children showed an association between prenatal antibiotic exposure, atopic dermatitis, and food allergies [40].

A systematic review from 2020 revealed that antibiotic prophylaxis for GBS diminishes beneficial commensals and profoundly influences the intestinal microbiota of the newborn, thus impacting the development of the immune system [31,40].

The potential advantages and adverse effects of probiotics in pregnancy have not been sufficiently explored so far. Apart from balancing the vaginal microbiome and preventing infection, probiotics have been shown to improve insulin resistance (reducing the risk of gestational diabetes), positively influence brain activity (leading to decreases in depression and anxiety), and enhance anti-inflammatory cytokines. They may also decrease the infants’ allergic reactions by downregulating Th2 responses [41,42].

According to the guidelines set forth by the National Institute for Health and Care Excellence (NICE), the administration of intrapartum antibiotics is recommended for specific groups of women during labor. Firstly, women in pre-term labor should receive intrapartum antibiotics as a preventive measure. Additionally, women who exhibit group B streptococcal colonization, bacteriuria, or infection during the current pregnancy should also receive intrapartum antibiotics. This recommendation extends to women who have had previous pregnancies with evidence of group B streptococcal colonization, bacteriuria, or infection unless they have obtained a negative test result for group B streptococcus via rectovaginal swab samples collected between 35 and 37 weeks of gestation or 3 to 5 weeks before the anticipated delivery date in the current pregnancy. Furthermore, women who have had a previous neonate diagnosed with an invasive group B streptococcal infection are included in this recommendation, as well as those diagnosed with chorioamnionitis. In cases of suspected early-onset infection, the guideline suggests the use of intravenous benzylpenicillin with gentamicin as the preferred antibiotic regimen for empirical treatment [43].

Different studies reveal different transmission rates of ESBL-positive bacteria (up to 48%) and MRSA (27.8%, 53.6%, 68%, and 80%) [19]. Because of the limited efficacy of empiric antibiotic treatment, sepsis with MDR bacteria is associated with increased morbidity and mortality [44]. Treatment options for neonatal infections caused by MDR bacteria are limited [45].

It has been estimated that by 2030, a third of the deliveries worldwide will be performed by cesarean section. Although the WHO recommends a single dose of pre-operative prophylactic antibiotic for cesarean sections and supports the use of narrow-spectrum antibiotics for this purpose, in many parts of the world women who undergo cesarean sections are recommended prolonged antibiotic courses [39].

An important aspect to be mentioned is that cesarean sections, one of the most commonly performed surgical procedures with approximately three times higher maternal morbidity than vaginal delivery and with important maternal complications in the following pregnancies, disturb the microbiota of the neonate, a disruption that could be exacerbated by antibiotic use, even if the antibiotics are only administered to the mother. Furthermore, it has been proven that the magnitude of the neonatal microbiome disruption is similar when comparing intrapartum with postnatal antibiotic administration. Therefore, it is possible that ESBL-PB and/or CRB colonization is less of a cause and more of an effect—a marker of the dysbiosis caused by antibiotic exposure of the mother during pregnancy [39,46,47,48].

Antimicrobial resistance is a growing global health threat and newborns are one of the main risk groups, owing to their underdeveloped immunity and microbiome. Exposure of the neonates to bacteria during birth can result in their colonization. Although colonization does not necessarily lead to disease (i.e., clinically manifest infection), when the microorganisms involved in this process are resistant to antibiotics, they can predispose to drug-resistant infections, especially in premature and low-birthweight infants. Drug-resistant infections have been linked to 200,000 neonatal deaths per year [39].

It must be taken into account that the mother is not the only possible source of transmission. Hospital staff, colonized surfaces, and visitors could be sources of neonate colonization. In the absence of risk factors, the screening of pregnant women or healthy newborns does not seem necessary if appropriate hygiene measures are respected [48,49].

### The Limitations of the Study

The laboratory methods employed did not allow for the detection of *Chlamydia* spp. and *Mycoplasma* spp., which are also known to cause abortions and pregnancy complications.

## 5. Conclusions

Aerobic bacteria originating from the gastrointestinal tract are frequent in cervical samples, and their antimicrobial resistance profiles can vary significantly. Understanding these resistance patterns is beneficial for assessing the potential implications during pregnancy or transmission to the newborn during birth, which may result in early neonatal infections. Therefore, appropriate supervision and treatment may be necessary to mitigate the risk of illness in newborns.

## Figures and Tables

**Table 1:** Microorganisms and Antimicrobial Resistance Patterns in Non-Cervical Clinical Samples.

Sample (N)	Microorganism (N)	Antimicrobial Resistance
Urine (23)	*E. coli* (15)	1 CSase1 MDR (R-FQ + R-SXT) 1 R-SXT
	*Enterococcus faecalis* (4) *Enterococcus faecium* (1)	isolated resistances, without therapeutic implications
	*K. pneumoniae* (2)	1 PDR *K. pneumoniae* (resistant to all tested antibiotics) 1 MDR (ESBL + R-FQ)
	*S. saprophyticus* (1)	isolated resistances, without therapeutic problems
Amniotic fluid (3)	*S. epidermis* (1)	sensitive to tested antibiotics, without therapeutic problems
	*K. pneumoniae* (1)	sensitive to tested antibiotics, without therapeutic problems
	*Enterococcus faecalis* (1)	sensitive to tested antibiotics, without therapeutic problems
Wound secretion (3)	*S. aureus* (1)	MDR (MRSA + R-FQ + R-SXT)
	*E. coli* (1)	R-FQ + R-SXT
	*Klebsiella pneumoniae* (1)	sensitive to most tested antibiotics, without therapeutic problems
Peritoneal cavity fluid (1)	*S. anginosus* (1)	sensitive to all tested antibiotics
Haemoculture (1)	*E coli* (1)	sensitive to tested antibiotics
Sputum (1)	*K. pneumoniae* (1)	XDR (ESBL + CRE + R-FQ + R-AG), sensitive to colistin

Abbreviations: CSase = cephalosporinase, MDR = multi-drug resistant, R-FQ = resistance to fluoroquinolones, R-SXT = resistance to trimethoprim-sulfamethoxazole, PDR = poly-drug resistance, MRSA = methicillin-resistant *S. aureus*, XDR = extensive drug resistance, ESBL = extended spectrum beta-lactamase, CRE = carbapenem resistant Enterobacteriaceae, R-AG = resistance to aminoglycosides.

**Table 2:** Clinical background of microbial isolates from cervical samples.

Microorganism	Group B Streptococcus	*E. coli*	*K. pneumoniae*	*S. aureus*
Abortion	25 (23.36%)	12 (15.79%)	5 (16.67%)	7 (31.82%)
Premature birth	9 (8.41%)	11 (14.47%)	5 (16.67%)	4 (18.18%)
Ectopic pregnancy	1 (0.94%)	5 (6.58%)	1 (3.33%)	-
Pathological pregnancy	5 (4.67%)	3 (3.95%)	-	1 (4.55%)
Maternal diseases	-	5 (6.58%)	-	1 (4.55%)
Monitored/full-term pregnancy	67 (62.62%)	40 (52.63%)	19 (63.33%)	9 (40.90%)
Total	107	76	30	22

Abbreviations: GBS = group B Steptococcus.

## Data Availability

Further information concerning the present study is available from the corresponding author upon reasonable request.
